# Netrin-1 Expression Is an Independent Prognostic Factor for Poor Patient Survival in Brain Metastases

**DOI:** 10.1371/journal.pone.0092311

**Published:** 2014-03-19

**Authors:** Patrick N. Harter, Jenny Zinke, Alexander Scholz, Julia Tichy, Cornelia Zachskorn, Hans M. Kvasnicka, Benjamin Goeppert, Céline Delloye-Bourgeois, Elke Hattingen, Christian Senft, Joachim P. Steinbach, Karl H. Plate, Patrick Mehlen, Dorothea Schulte, Michel Mittelbronn

**Affiliations:** 1 Edinger Institute, Institute of Neurology, University of Frankfurt am Main, Frankfurt am Main, Germany; 2 German Cancer Consortium (DKTK), Heidelberg, Germany; 3 German Cancer Research Center (DKFZ), Heidelberg, Germany; 4 Senckenberg Institute of Neurooncology, University of Frankfurt am Main, Frankfurt am Main, Germany; 5 Senckenberg Institute of Pathology, University of Frankfurt am Main, Frankfurt am Main, Germany; 6 Institute of Pathology, University of Heidelberg, Heidelberg, Germany; 7 Apoptosis, Cancer and Development Laboratory, Equipe labellisée ‘La Ligue’, LabEx DEVweCAN, Centre de Cancérologie de Lyon, INSERM U1052-CNRS UMR5286, Université de Lyon, Centre Léon Bérard, Lyon, France; 8 Institute of Neuroradiology, University of Frankfurt am Main, Frankfurt am Main, Germany; 9 Department of Neurosurgery, University of Frankfurt am Main, Frankfurt am Main, Germany; National Cancer Center, Japan

## Abstract

The multifunctional molecule netrin-1 is upregulated in various malignancies and has recently been presented as a major general player in tumorigenesis leading to tumor progression and maintenance in various animal models. However, there is still a lack of clinico-epidemiological data related to netrin-1 expression. Therefore, the aim of our study was to elucidate the association of netrin-1 expression and patient survival in brain metastases since those constitute one of the most limiting factors for patient prognosis. We investigated 104 brain metastases cases for netrin-1 expression using in-situ hybridization and immunohistochemistry with regard to clinical parameters such as patient survival and MRI data. Our data show that netrin-1 is strongly upregulated in most cancer subtypes. Univariate analyses revealed netrin-1 expression as a significant factor associated with poor patient survival in the total cohort of brain metastasis patients and in sub-entities such as non-small cell lung carcinomas. Interestingly, many cancer samples showed a strong nuclear netrin-1 signal which was recently linked to a truncated netrin-1 variant that enhances tumor growth. Nuclear netrin-1 expression was associated with poor patient survival in univariate as well as in multivariate analyses. Our data indicate both total and nuclear netrin-1 expression as prognostic factors in brain metastases patients in contrast to other prognostic markers in oncology such as patient age, number of brain metastases or Ki67 proliferation index. Therefore, nuclear netrin-1 expression constitutes one of the first reported molecular biomarkers for patient survival in brain metastases. Furthermore, netrin-1 may constitute a promising target for future anti-cancer treatment approaches in brain metastases.

## Introduction

Beside its role as a major axonal guidance molecule in mammalian CNS development, netrin-1 also exerts anti-apoptotic effects via binding to one of its major dependence receptors, deleted in colorectal cancer (DCC), thereby driving tumorigenesis [1], [2]. There is also evidence that netrin-1 enables an increased supply of oxygen and nutrients to tumor cells by the formation of new blood vessels especially in the context of pancreatic adenocarcinomas [3]. In addition, netrin-1 acts as a pro-angiogenic factor most probably by inhibiting endothelial cell apoptosis through UNC5B and activating a DCC-dependent ERK-1/2-eNOS feed-forward mechanism [4]–[7]. In contrast, other reports indicate that netrin-1 leads to inhibition of endothelial cell sprouting during development [8]. The involvement of netrin-1 in the spread of cancer cells was corroborated by the finding that especially metastatic breast carcinomas show netrin-1 overexpression compared to their non-metastatic counterparts [9]. However, since netrin-1 is strongly overexpressed in various cancer entities, it has been presented as a pluripotent tumor biomarker independent of the grade of malignancy or tumor subtype [10]–[12]. For some cancer subtypes such as adenocarcinomas of the pancreas, it was demonstrated that netrin-1 is either associated with significantly earlier relapse or worse patient overall survival [13]. More recently, the malignant potential of secreted full-length netrin-1 has been extended to a truncated intranucleolar variant, ΔN-netrin-1, which also induces tumor cell growth [14]. It remains to be determined to which extent the frequently observed methylation changes or loss of netrin-1 receptors, among others DCC and members of the UNC5 family, may contribute to the increased malignant potential of tumor cells [15]–[19]. First preclinical studies revealed that the inhibition of netrin-1 interaction with its receptors by a decoy recombinant DCC fragment (DCC-5Fbn) led to increased tumor cell death and significant reduction of the tumor mass in a murine xenograft lung cancer model [20], [21]. Tumor cells metastasizing to the CNS constitute one of the most detrimental events for patient prognosis. However, to date, no data are available concerning the expression of netrin-1 in brain metastases and its association with patient survival. Therefore, the aim of our study was to investigate netrin-1 expression in a large cohort of brain metastasis and to analyze its suitability as a prognostic marker serving as a basic rationale for future anti-netrin-1 treatment approaches in secondary brain tumors.

## Materials and Methods

### Clinical Data and Tissue Specimens

We generated tissue micro arrays (TMAs) from paraffin-embedded tissue specimens from the human tissue brain bank of the Edinger Institute (Neurological Institute), Goethe University Frankfurt, Germany, member of the German Consortium for Translational Cancer Research (DKTK), Heidelberg, Germany **(** **Table 1** **)**. The study protocol was endorsed by the local ethical committee of the Goethe University Frankfurt, Germany (GS 4/09; SNO_01-12). Survival data could be recorded for 76 patients starting from day of neurosurgical intervention (brain metastasis resection). The amount of edema as well as the number of brain metastases was assessed using T1-weighted MRI images with contrast medium and corresponding T2-weighted images. For quantification of edema we applied a semi-quantitative score (0: no edema; 1: small edema margin around tumor bulk; 2: locally restricted edema; 3: extensive edema.).

**Table 1:** Overview about the brain metastasis cohort constituting a representative sample compared to previously published cohorts [38], [39].

*Brain metastases*	*n*	*% of all metastases*	*mean survival (days)*	*mean survival 95% CI*
NSCLC	33	31.7	321	175 to 468
SCLC	7	6.7	277	0 to 558
Breast carcinoma	19	18.3	594	360 to 829
Melanoma	11	10.6	499	69 to 930
Renal cell carcinoma	10	9.6	858	239 to 1477
Colon carcinoma	7	6.7	574	83 to 1066
Carcinoma NOS	11	10.6	158	35 to 282
Others	6	5.8	394	0 to 787
TOTAL	104	100	450	349 to 551

The group of “others” consists of few cases of carcinomas deriving from uterus, cervix, esophagus and a single case of leiomyosarcoma. (NOS: not otherwise specified).

### Immunohistochemistry

All samples were fixed in 4% formaline (pH 7.4) and embedded in paraffin. For deparaffination and rehydration we used standard procedures with alcohol and xylene. Slides were cut at a microtome (3 μm), immunohistochemistry was performed using the automated Ventana Discovery XT staining system (Ventana, Tucson, Arizona USA) and standard protocols. We used rabbit anti-netrin-1 (Santa Cruz Biotechnologies, Santa Cruz, USA, clone H-104, dilution 1∶20) and mouse anti-DCC antibodies (Leica/Novocastra, Newcastle upon Tyne, UK, Clone DM51, diluted 1∶20). Antibody specificity has been shown before [22]. Ki67 immunohistochemistry was performed according to standard diagnostic protocols on the Discovery XT immunohistochemistry system (Ventana, Strasbourg, France) using the monoclonal mouse anti-human Ki67 antibody (dilution 1∶200, Clone MIB-1, Dako, Glostrup, Denmark).

### In-situ Hybridization

Whole mount sections and TMAs were investigated for netrin-1 mRNA expression by in-situ hybridization. For this purpose, human cDNA for netrin-1 was cloned in a pcDNA3.1 plasmid and Sp6 and T7 polymerases were used for the generation of Digoxygenin-labeled sense and antisense probes, respectively [20]. Paraffin embedded sections were rehydrated by a descending alcohol series and boiled for 40 min in citrate buffer (1.05 g citric acid in 500 ml deionised water, pH 6,0), then allowed to cool down for 20 min. Endogenous alkaline phosphatase was inactivated with 0.2 M HCL. The slides were washed in PBS and proteins were denatured by proteinase K (10 μg/ml) solved in Tris-EDTA (TE) buffer. For the inactivation of proteinase K, slides were washed in 0.1M glycin and rinsed in PBS. 625 μl acetic anhydride in 250 ml 0.1M triethanolamine was used for acetylation. For prehybridization, slides were incubated in hybridization solution consisting of 50% de-ionized formamide, 4x standard saline citrate, 5% dextransulfate (w/v), 5x Denhardt’s, 0.1% SDS, 0.5 mg/ml yeast t-RNA (10 mg/ml) and 0.25 mg/ml Salmon Sperm DNA for 5 h at room temperature. The Digoxygenin-labeled probes were first diluted in DEPC-water, then in hybridization solution to obtain the final concentration of 1 ng/μl followed by denaturation at 95°C. Hybridisation at 65°C was performed overnight. The next day, slides were first rinsed in 0,2x standard saline citrate at 70°C and then in 2x standard saline citrate at room temperature. Dig-1 (for 5xDig-1∶0.5M maleic acid diNa diH_2_O, 0,75M NaCl ad 1l water,pH 7,5) incubation was followed by blocking in Dig-2 (10% blocking reagent Boehringer (10% w/v) in Dig-1) for 1 h at room temperature. Slides were incubated with anti-Dig antibody diluted (1∶500) in Dig-2 for 1 h, then rinsed three times in Dig-1 followed by one rinse in Dig-3 (0,1M Tris base, 0,1M NaCl, 0,05M MgCl_2_). Finally, the slides were incubated in staining solution (10% polyvenyl alcohol, Dig-3, Nitro blue tetrazolium chloride (NBT), 5-Bromo-4-chloro-3-indolyl phosphate (BCIP)) for 3,5 h at 30°C, and rinsed in PBS. Slides were counterstained with hematoxylin for 20 sec and mounted in Aquatex.

### Scoring

Three independent investigators (two trained and experienced neuropathologists (PNH and MM) and one briefly trained inexperienced developmental biologist (DS)) analyzed netrin-1 expression assessing staining intensity and frequency using a semi-quantitative score. The frequency score ranged from 0–4; meaning 0∶0–1%, 1∶1–10%, 2∶10–25%, 3∶25–50% and 4: >50% of all cells showing a positive staining. Likewise, intensity was recorded in a similar semiquantitative approach as follows; 0: no staining, 1: weak staining, 2: moderate staining, 3: strong staining. The two results for staining intensity and frequency were multiplied, so that the “tumor cell score” reflected both [22] (DCC scoring followed the same protocol). Additionally, we scored the percentage of cells with nuclear netrin-1 expression and Ki67-positive cells as a fraction of all cells counted. The evaluation and photographic documentation of the immunohistochemical staining was performed using an Olympus BX50 light microscope.

### Statistical Analyses

Scoring data is given as box-plots in the figures. Correlation of netrin-1 and DCC expression as well as correlation of nuclear netrin-1 and Ki67 expression was assessed using a pairwise analysis with subsequent non-parametric Spearman’s ρ testing. The association of patient survival with the response variable (tumor cell score, nuclear netrin-1 as well as Ki67 expression) was analyzed by Kaplan-Meier analysis tested by log-rank and Wilcoxon test. Netrin-1 expression levels were dichotomized at the median and referred to as low (score<or = 4) or high (score >4) expression levels. Kaplan-Meier analysis on nuclear netrin-1 levels was performed categorizing low nuclear netrin-1 (< or = 5%) and high nuclear netrin-1 (>5%) frequencies also after dichotomization at the median. Dichotomization of DCC scores classified scores<or = 9 as low and scores >9 as high. Multivariate analysis was performed using the Cox proportional hazard model controlling for nuclear netrin-1 expression (positive cells/all tumor cells), Ki67 proliferation rate (positive cells/all tumor cells), diagnosis, patient age, number of brain metastases and edema score. A significance level of alpha = 0.05 was chosen for all testings. Statistical analysis was performed using JMP 8.0.1 software (SAS, Cary, NC, USA).

## Results

### Netrin-1 is Strongly Expressed in Human Brain Metastases

**Figure 1:**
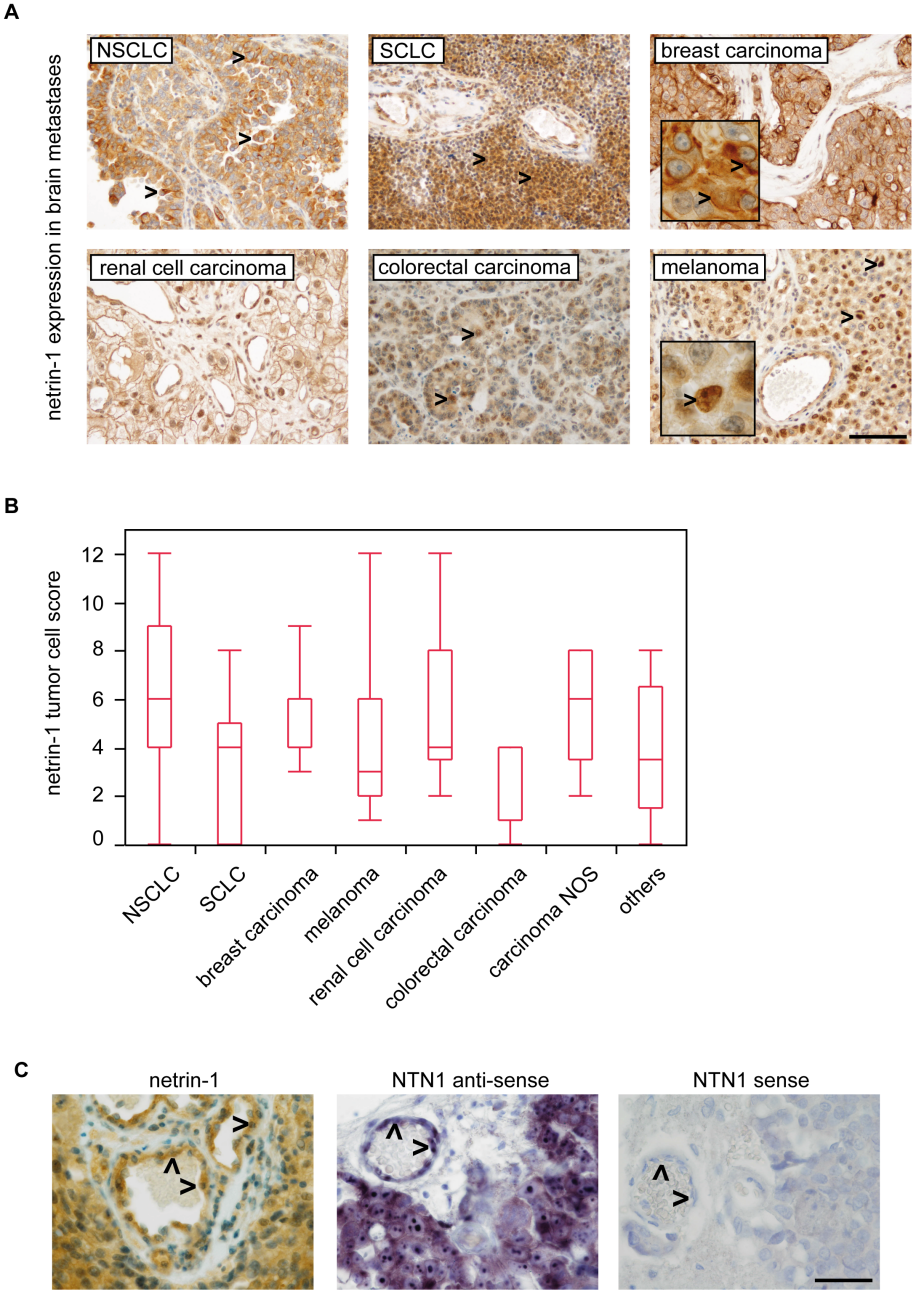
Netrin-1 is strongly expressed in tumor cells of human brain metastases. Arrowheads in (A) indicate different subcellular localisation. Brain metastases of NSCLC and SCLC show strong cytoplasmic staining, while the depicted breast carcinoma shows an additional membraneous signal (see blow up and arrowheads). Nuclear localization is exemplarily shown in melanoma (see blow up and arrowhead) and colon cancer metastases samples (scale bar 100 μm). Box plots of netrin-1 tumor cell scores are shown in (B) (see also **Table S1**). In-situ hybridization reveals tumor cells and endothelial cells (arrowheads) as source for netrin-1 expression (C: example of a SCLC, scale bars 50 μm).

**Figure 2:**
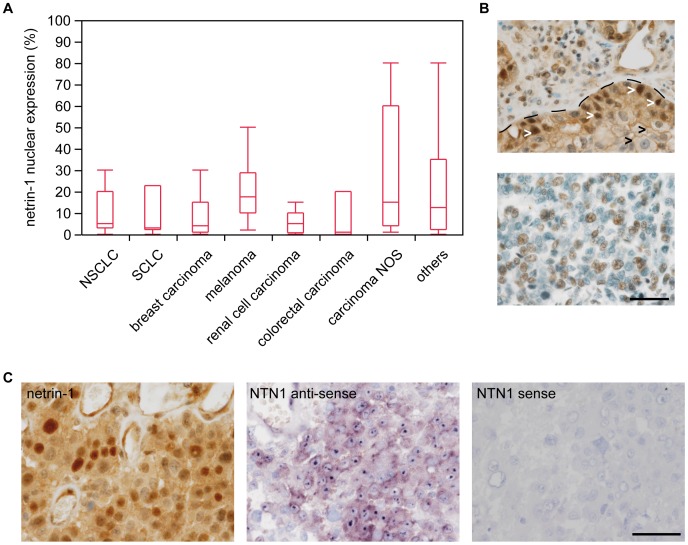
Nuclear localization of netrin-1 in brain metastases. (A) Box plots showing different expression profiles of nuclear netrin-1 in brain metastases of the tumor types indicated (see also **Table S1**). (B, upper) Example of NSCLC with strong cytoplasmic and nuclear netrin-1 expression. Dotted line shows tumor to mesenchymal stroma border with a regionally heterogenous netrin-1 expression pattern. While tumor cells adjacent to the stroma show strong nuclear staining, tumor cells inside the tumor nodule exhibit weaker netrin-1 expression without showing a nuclear localization. (B, lower) Brain metastases of breast carcinoma exclusively showing nuclear netrin-1 expression (scale bar 50 μm). (C) Comparison of netrin-1 protein expression and mRNA levels in an exemplaric case of melanoma brain metastasis (scale bar 50 μm). While netrin-1 protein can be detected in cytoplasms but stronger in nuclei, in-situ hybridization reveals a mild cytoplasmic staining compared to stronger signals within the nucleus.

Immunohistochemical analysis as well as in-situ hybridization showed that netrin-1 is strongly expressed in human brain metastases. We found a strong netrin-1 upregulation especially in brain metastases of lung carcinomas (NSCLC and SCLC) as compared to normal lung tissue (**Figure S1**). Comparing different subtypes of brain metastases, highest netrin-1 levels were detected in secondary brain tumors deriving from the lung followed by breast carcinomas and carcinomas which were not otherwise specified (NOS) **(** **Figure 1 A and B** **; Table S1)**. Interestingly, brain metastases deriving from colorectal tumors showed only minor netrin-1 expression. Netrin-1 was mainly localized in tumor cell cytoplasms, bound to tumor cell membranes or found within tumor cell nuclei **(** **Figure 1 A)**. Inter-rater correlation of the scoring was strong (r>0.7 and p<0.0001 in Spearman’s ρ testing of 3 independent investigators). In addition to the tumor cell-associated netrin-1 expression, also some stromal and endothelial cells were strongly netrin-1-positive. In-situ hybridization revealed that endothelial cells are also a major source for netrin-1 expression **(** **Figure 1 C** **)**. The comparison of immunohistochemical stainings and in-situ hybridization revealed a similar netrin-1 expression profile **(Figure S1)**. As recently described a truncated ΔN-netrin-1 isoform regulating tumor cell proliferation of neuroblastoma cells is localized to the nucleus and nucleolus [14]. In our samples we detected nuclear netrin-1 in all different brain metastasis subentities with highest frequencies in melanomas and carcinomas NOS **(** **Figure 2 A, B** **)**. Most of the samples showed both a nuclear and cytoplasmic signal, however we observed an exclusive nuclear expression in some cases **(** **Figure 2 B** **)**. Additionally, we focally observed a regional hierarchy of nuclear netrin-1 signal, which was most prominently seen adjacent to the fibrovascular tumor stroma **(** **Figure 2 B** **)**. Interestingly, samples with a strong nuclear staining for netrin-1 also showed a strong nuclear/nucleolar signal in the in-situ hybridization analyses **(** **Figure 2 C** **)**. In addition to immunohistochemical stainings and in-situ hybridizations we exemplarily analyzed cryo-material of human brain metastases for netrin-1 expression using qRT-PCR, western blot and immunohistochemistry of the corresponding frozen section. We were able to detect both forms, the high molecular full length netrin-1 and the truncated ΔN-netrin-1 in western blot analysis (**Figure S2**). Quantification of protein levels as well as RNA roughly correlated with immunohistochemistry (**Figure S2**).

### Netrin-1 is Associated with Poor Patient Survival in Brain Metastases

**Figure 3:**
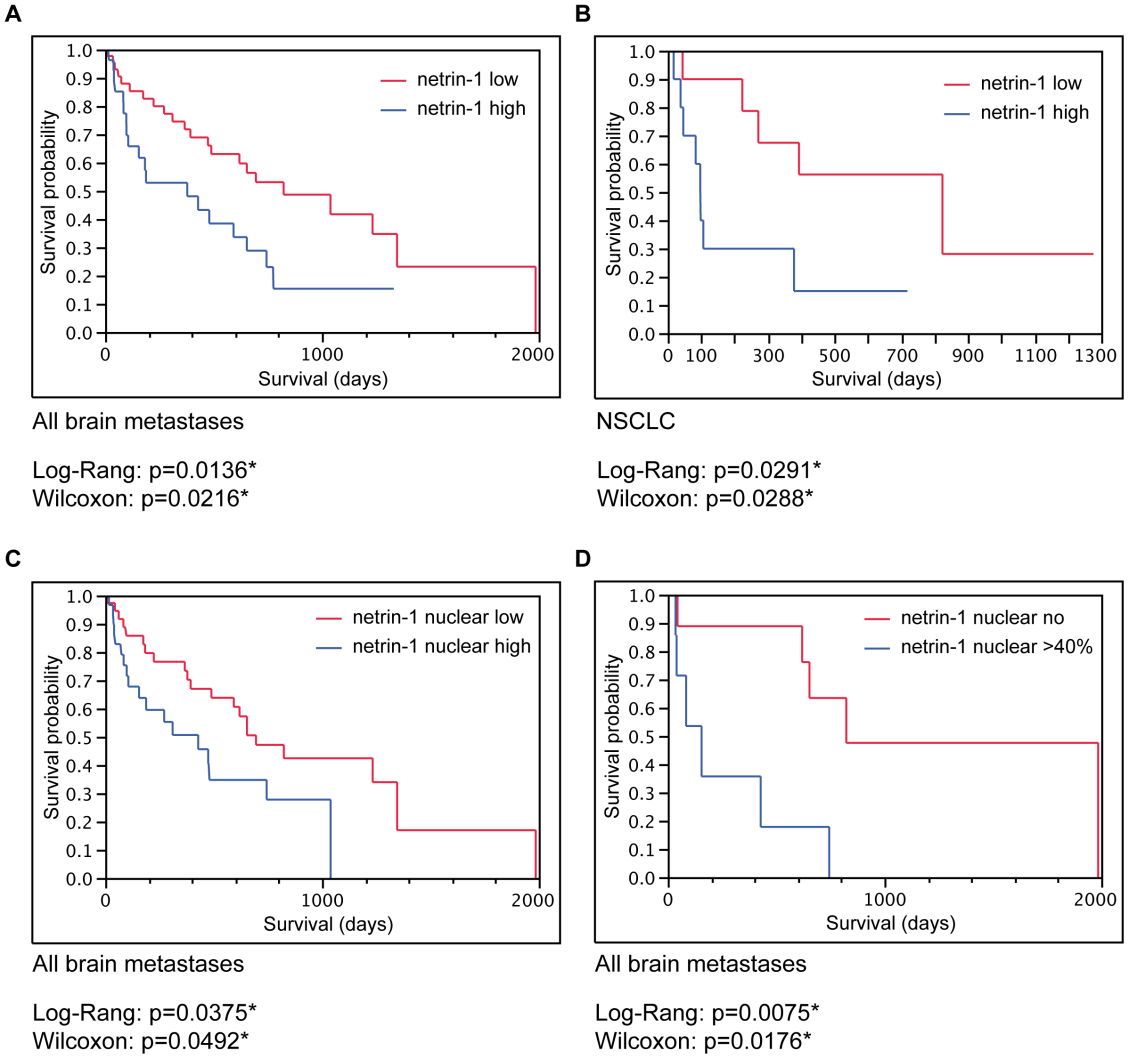
Univariate survival analysis for total netrin-1 and nuclear netrin-1 expression. Univariate Kaplan-Meier survival analysis showing poor prognosis for high netrin-1 tumor cell scores in patients with brain metastases. Survival probability of (A) all brain metastases (entire cohort) and (B) the subentity of NSCLC. (C, D) Kaplan-Meier survival analysis showing worse survival in brain metastases patients when netrin-1 locates to the nucleus in (C) the total cohort and (D) subgroups comparing patients without nuclear netrin-1 to the cohort exhibiting highest nuclear netrin-1 levels. (Low netrin-1 score<or = 4 or high netrin-1 score >4; low nuclear netrin-1< or = 5% or high nuclear netrin-1>5%.).

To test the potential association of netrin-1 expression levels with survival in patients with brain metastases, we first performed a median split of all cases according to their netrin-1 levels (low versus high netrin-1 levels). Univariate Kaplan-Meier analysis revealed high netrin-1 levels as a factor associated with poor patient survival in our cohort of all brain metastases **(** **Figure 3 A** **)**. Additionally, also the subgroup of NSCLC brain metastases demonstrated significantly reduced patient survival rates in case of high netrin-1 levels in tumor cells **(** **Figure 3 B** **)**. These effects on patient survival were not seen for netrin-1 receptor DCC **(Figure S3)**. Correlation analysis revealed netrin-1 and DCC as independently expressed in all brain metastases (p = 0.2828; Spearman’s ρ = 0.1119; data not shown).

### Nuclear Netrin-1 Expression is an Independent Prognostic Marker in Brain Metastases

Further we analyzed the association of nuclear netrin-1 expression and its impact on patient survival. Median split and subsequent Kaplan-Meier survival analysis revealed worse patient survival in case of high nuclear netrin-1 expression frequencies **(** **Figure 3 C** **)**. Additionally, we analyzed patients with absence of nuclear netrin-1 expression and compared them to the patient group showing highest nuclear netrin-1 expression (>40% of tumor cells). Survival analyses exhibited even poorer survival probability in the group of highest expression levels compared to brain metastases without nuclear netrin-1 expression **(** **Figure 3 D** **)**.

### The Ki67 Proliferation Rate is Associated with Poor Patient Survival in Brain Metastases Independent of Nuclear Netrin-1 Expression

**Figure 4:**
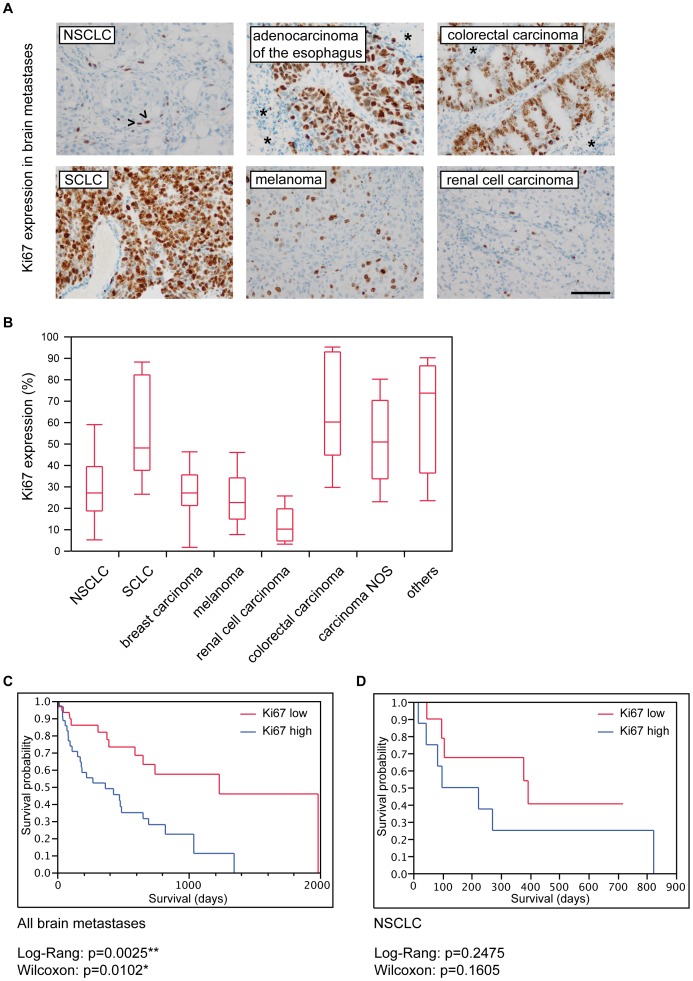
The Ki67 proliferation rate is only significantly associated with patient survival in univariate analysis. (A) Ki67 expression in different brain metastases. We focally observed proliferative activity predominantly in tumor cells adjacent to the fibrovascular stroma (see arrowheads in NSCLC). Highly necrotic tumors (see asterisks in adenocarcinoma of the esophagus and colorectal carcinoma) showed Ki67 expression predominantly in vital tumor parts surrounding the fibrovascular stroma. Brain metastases of SCLC, colorectal carcinomas and carcinomas NOS most often presented with highest Ki67 indices, whereas renal cell carcinomas and melanomas showed a lower proliferative activity (A and B; scale bar 100 μm). (C) Univariate Kaplan-Meier survival analysis revealing significantly reduced survival probability in patients with high as compared to low Ki67 levels. (D) In subentities such as NSCLC no significant differences were detected.

As the truncated nuclear or nucleolar ΔN-netrin-1 isoform has been linked to the regulation of cell proliferation in tumor cells, we tested our cohort for a potential correlation of nuclear netrin-1 and Ki67 proliferation index. Although strong regional differences were observed for Ki67 **(** **Figure 4 A** **)**, no correlation with nuclear netrin-1 expression could be detected (p = 0.8662; Spearman’s ρ = 0.018; data not shown). The Ki67 proliferation index considerably differed between cancer subtypes showing highest levels in carcinomas NOS and colorectal cancers and lowest levels in renal cell carcinomas **(** **Figure 4 B** **)**. Concerning the prognostic impact of the Ki67 proliferation rate in brain metastases, univariate Kaplan-Meier survival analysis revealed a significant association of high Ki67 levels with poorer patient survival in our total cohort **(** **Figure 4 C** **)** while in subentities like NSCLC no significant prognostic values were found for Ki67 **(** **Figure 4 D** **)**.

### Nuclear Netrin-1 Expression is an Independent Prognostic Marker for Brain Metastases in Multivariate Survival Analyses

To further assess the clinico-pathological relevance of our findings, nuclear netrin-1 expression (positive cells/all tumor cells) was analyzed in a multivariate Cox proportional hazard model controlling for diagnosis, patient age (in years), number of brain metastases, amount of edema (edema score) and Ki67 proliferation rate (positive cells/all tumor cells). Most remarkably, multivariate analysis revealed nuclear netrin-1 expression as an independent prognostic factor associated with poor patient survival in brain metastases together with the diagnosis of the brain metastasis subtype **(** **Table 2** **)**. In contrast, factors which are frequently associated with patient survival in oncological studies in general such as patient age, number of brain metastases or tumor cell proliferation rate were not significantly related to patient prognosis in the multivariate analysis.

**Table 2:** Multivariate survival analysis including all brain metastasis patients.

*Variable*	*Results of multivariate Cox regression*	
	p-Value	Hazard Ratio (95% CI)
Diagnosis	0.0030	n/a
Patient age	0.0516	1.04 (0.99–1.08)
Number of brain metastases	0.1428	1.08 (0.97–1.20)
Edema score	0.1912	n/a
Ki67 (%)	0.3966	1.01 (0.98–1.04)
Netrin-1 nuclear (%)	0.0051	1.04 (1.01–1.07)

Cox hazard ratios of nuclear netrin-1, Ki67, patient age and number of brain metastases are depicted (single comparisons of diagnoses and edema scores are not shown. n/a not applicable). n/a: not assessed.

## Discussion

Brain metastases constitute one of the most detrimental life-threatening condition in humans and are associated with poor survival reaching median overall survival times of 10.7 months in renal cell carcinomas or 18 months in non-small cell lung cancer treated with stereotactic radiosurgery [23], [24]. However, these data obtained from metastasis subgroups are somehow in contrast to larger series of unselected patient cohorts in which the median overall survival remained relatively stable over the last 4 decades reaching only between 4–6 months [25], [26]. The survival time is strongly related to the therapeutic approach. In a cohort of brain metastasis deriving from colorectal cancer, the median overall survival ranged from 1 month in case of steroid application only, over 3 months if radiotherapy was applied to 9 months for the subgroup that underwent surgical resection [27]. To date only very few molecular factors are known to be significantly associated with a better patient survival in brain metastasis cohorts, such as an EGFR mutation in non-small cell lung cancers [28]. Therefore, there is lack of knowledge about molecular markers which are associated with patient survival once metastases are already established in the brain parenchyma. In our study, netrin-1 was strongly expressed in brain metastases of different primary tumors with highest levels in brain metastases of NSCLC and carcinomas which were not otherwise specified. Interestingly, colorectal carcinomas showed lowest netrin-1 levels, which is corroborated by publicly available databases on primary colorectal carcinomas (data not shown). Following the concept of the dependence receptor function tumor cells might decide whether they select for loss of netrin-1 receptors as for instance in colorectal cancers or select for an upregulation of netrin-1. The exact underlying mechanisms of low netrin-1 levels in metastatic colorectal carcinomas and consequent effects are not understood and are still under debate [9].

Most strikingly we were able to establish netrin-1 protein expression as one of the first molecular markers for poor survival in a large patient cohort suffering from brain metastases from different primary tumors. The median survival time was only about 5 months (172 days; range 6–1331) in the group showing high netrin-1 expression in brain metastases while patients with low netrin-1 levels had a median life expectancy of more than one year (476 days; range 7–1985). Interestingly, when we specifically analyzed nuclear netrin-1 localization, we even found stronger significant differences in patient survival with a mean survival time of 595 days (range 7–1985) for low nuclear expression compared to a mean survival time of only 161 days (range 6–1039) for high nuclear netrin-1 expression. Beside the pathological diagnoses of the brain metastasis subtype, nuclear netrin-1 expression was also an independent prognostic factor for patient survival, while other frequently assessed prognostic oncological variables such as patient age, number of brain metastases [29] or even the proliferative capacity of tumor cells (Ki67 index) were not. This multivariate analysis included all brain metastases from different primary tumors. As the whole cohort was composed of varying patient size (e.g. NSCLC patients n = 33; melanoma patients n = 11; renal cell carcinoma patients n = 10), one could assume that the group of NSCLC patients might mask the smaller groups. Therefore, we excluded the NSCLC patients from our analysis and performed another multivariate analysis, which revealed nuclear netrin-1, pathological diagnosis and number of brain metastases as independent prognostic factors while Ki67 proliferation rate and patient age were still no prognostic factors (see **Table S2**). The fact that patient age is not prognostic in our cohort might be contra-intuitive at first glance as patient age is frequently a prognostic factor in human cancers. Nevertheless, the patients in our study are already pre-selected as they were operable. Since many patients with brain metastases who are in a worse clinical condition would not be operated but only accompanied by palliative care, our cohort might be more homogenous as compared to population-based cohorts.

The truncated ΔN-netrin-1 variant which localizes to the nucleolus has been shown to affect ribosome biogenesis resulting in cancer cell proliferation [14]. Beside the nucleolar expression of netrin-1, we observed a strong nuclear localisation in tumor cells which indicates a nuclear function beyond ribosome biogenesis as for instance as transcription factor or cofactor regulating target gene transcription or similar to EGFR as DNA repair mechanism in response to chemotherapy and ionizing radiation [30]. The nuclear epidermal growth factor receptor (EGFR) function was also associated with (I) poor patient survival in breast cancer patients and (II) with increased local recurrence in oropharyngeal squamous cell cancer [31], [32]. Beside receptor-tyrosine kinases (RTKs) localizing to the nucleus thereby regulating cell cycle and target gene transcription [33], [34] even typically secreted factors such as fibroblast growth factor bFGF [35] have the ability to localize to the nucleus. Similarly to its function on membrane-associated receptors, EGF also autophosphorylates its intranuclear receptors or receptors which are bound to the nuclear membrane [36]. In the case of nuclear/nucleolar netrin-1 expression, it is surprising that no significant correlation with the Ki67 proliferation index could be observed since previous *in vitro* studies linked the intranuclear localisation of netrin-1 to an enhanced proliferative activity [14]. Our data might not only have prognostic but also therapeutic impact since netrin-1 is involved in tumorigenesis in general and treatment strategies against netrin-1 have already been successfully tested in preclinical trials [20], [21], [37]. However, it is not known whether the current preclinical anti-netrin-1 approaches, which mainly focus on trapping external netrin-1 to induce tumor cell apoptosis, can be modified in a way to block also the intracellular action of netrin-1.

In summary, our findings present the nuclear expression of netrin-1 as an independent prognostic biomarker for poor patient survival in human brain metastases. As most of the investigated molecular markers in brain metastases did not turn out to be significantly associated with patient survival to date, nuclear netrin-1 expression constitute one of the first biomarkers which can be easily applied in the daily diagnostic setting. The analysis of nuclear netrin-1 expression adds a valuable prognostic information since the absence of nuclear netrin-1 in brain metastases is associated with more than three times longer survival. Additionally, further studies have to elucidate if treatment strategies targeting nuclear netrin-1 might constitute a promising approach for the most devastating clinical condition of brain metastases.

## Supporting Information

Figure S1 **Netrin-1 is upregulated in brain metastases of NSCLC as compared to normal lung tissue.** (A) Netrin-1 is only weakly expressed in normal bronchial epithelium, however strongly upregulated in NSCLC brain metastases both on protein (left: immunohistochemistry) and mRNA (middle, right: in-situ hybridization) level (scale bar 100 μm). (B) NSCLC cells showing strong netrin-1 expression (left) as determined by immunohistochemistry (right: negative control with omission of the first antibody; scale bar 100 μm).
(TIF)

Figure S2 **Correlation of netrin-1 expression at mRNA and protein level in human brain metastases.** (A) Western blot showing full length netrin-1 protein, as well as a strong band for the nuclear ΔN-netrin-1 variant (abbreviation MNOF followed by numbers stands for anonymized patient samples of brain metastases). Recombinant FLAG-tagged netrin-1 served as a positive control showing higher molecular weight due to the FLAG-tag. Intensity measurements of either full length or ΔN-netrin-1 in association with alpha-tubulin loading control resulted in the depicted ratio. ImageJ software (NIH, USA) was used for quantification. (B) Corresponding cryo sections from the same tumor specimens were stained for netrin-1 by automated immunohistochemistry. Left row showing a representative tumor area, right row highlighting the nuclear compartment (scale bar left row 100 μm, scale bar right row 50 μm). (C) Corresponding qRT-PCR analyses from extracted mRNA of the same tumor specimens as assessed for protein expression levels is depicted. Netrin-1 (NTN1) expression relative to HPRT1 and G6PD1 housekeeping genes. We used a primer set overspanning intron 1 of the netrin-1 (NTN1) gene (forward primer: GCAAGCCCTTCCACTACGAC; reverse primer: CGACAGTTGAGGCAGACACCT).
(TIF)

Figure S3 **DCC expression in human brain metastases.** DCC is strongly expressed in tumor cells of human brain metastases and is mainly localized in a granular pattern in cytoplasms but also on cell membranes (A; scale bar 100 μm). Box plots of DCC tumor cell scores are shown in (B) (see also **Table S1**). (C, D) No association of DCC tumor cell scores with patient survival neither in (C) the entire cohort nor in (D) the subentity of NSCLC was found.
(TIF)

Table S1 **Median and mean expression levels of netrin-1 and DCC in human brain metastases.** Median tumor cell expression scores of netrin-1 and DCC and percentages of netrin-1-positive tumor cell nuclei (per total tumor cell nuclei) in human brain metastases.
(DOCX)

Table S2 **Multivariate survival analysis in our patient cohort excluding NSCLC.** Cox hazard ratios of nuclear netrin-1, Ki67, patient age and number of brain metastases are depicted (single comparisons of diagnoses and edema scores are not shown. n/a not applicable).
(DOCX)
