# Supplementary figures and images for: Netrin-1 Expression Is an Independent Prognostic Factor for Poor Patient Survival in Brain Metastases
Source: PLoS One. 2014 Mar 19;9(3):e92311. doi: 10.1371/journal.pone.0092311 (PMC3960244; doi:10.1371/journal.pone.0092311)

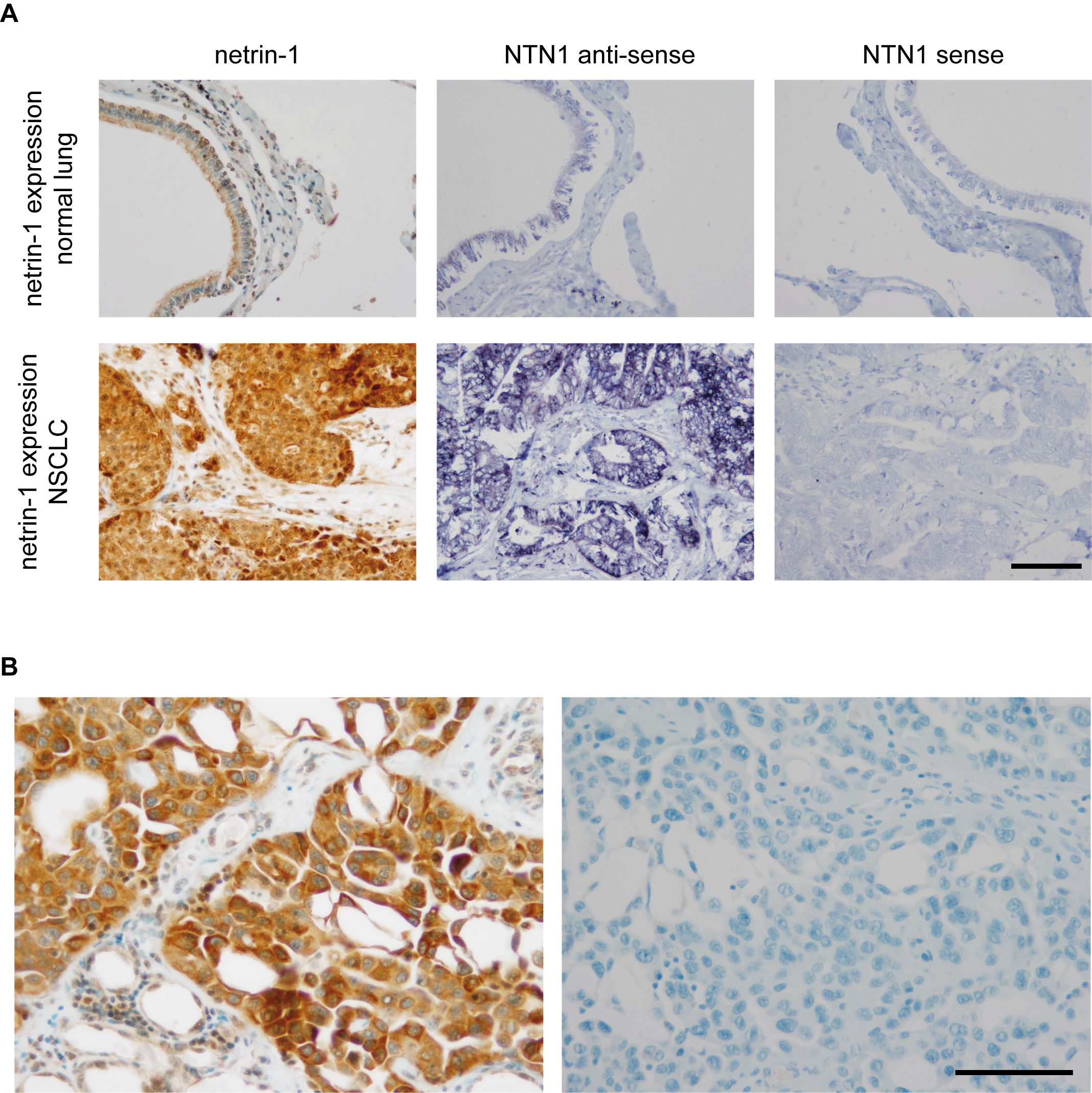

Supplement: Figure S1 — Netrin-1 is upregulated in brain metastases of NSCLC as compared to normal lung tissue. (A) Netrin-1 is only weakly expressed in normal bronchial epithelium, however strongly upregulated in NSCLC brain metastases both on protein (left: immunohistochemistry) and mRNA (middle, right: in-situ hybridization) level (scale bar 100 μm). (B) NSCLC cells showing strong netrin-1 expression (left) as determined by immunohistochemistry (right: negative control with omission of the first antibody; scale bar 100 μm). (TIF) [file pone.0092311.s001.tif]

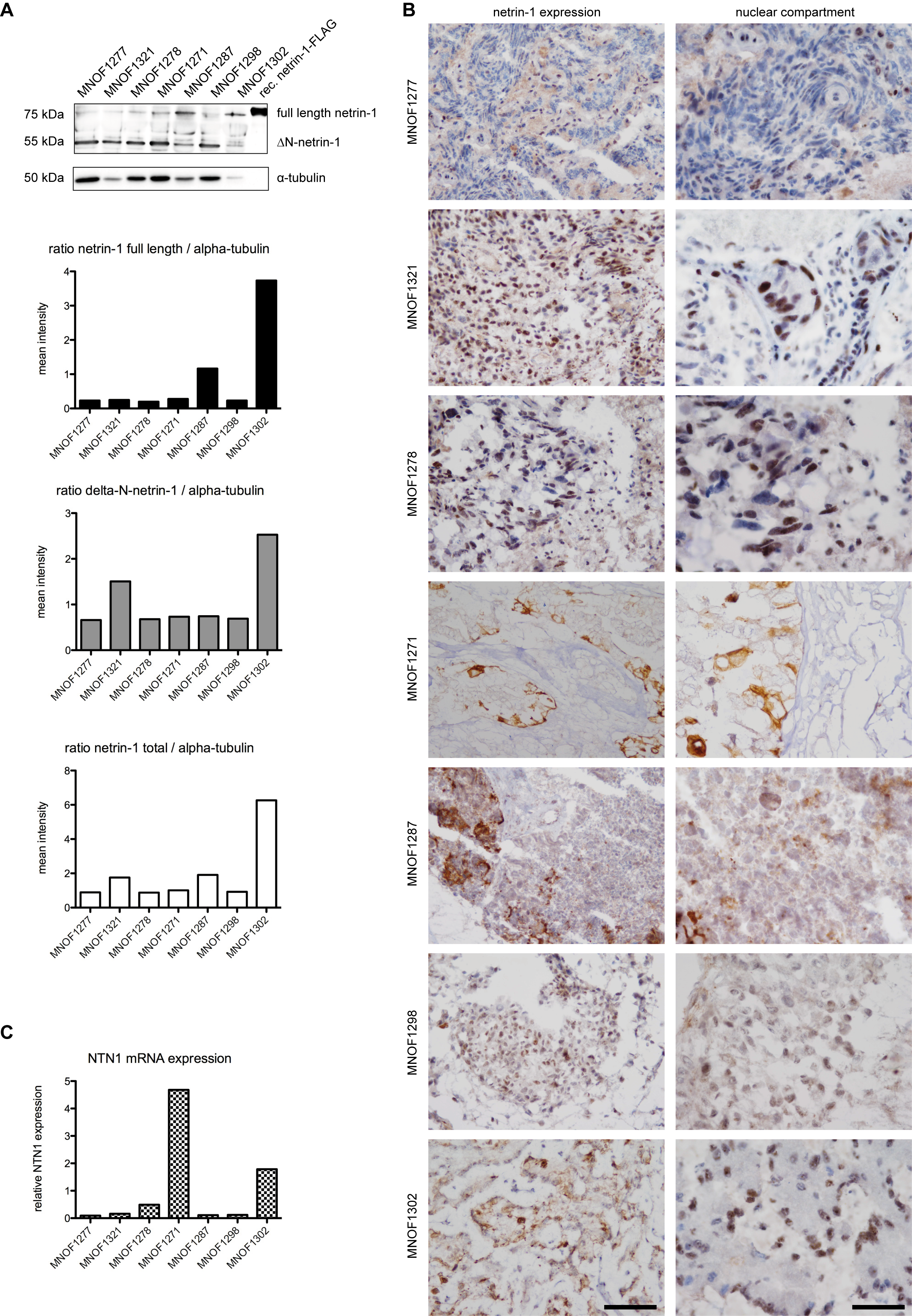

Supplement: Figure S2 — Correlation of netrin-1 expression at mRNA and protein level in human brain metastases. (A) Western blot showing full length netrin-1 protein, as well as a strong band for the nuclear ΔN-netrin-1 variant (abbreviation MNOF followed by numbers stands for anonymized patient samples of brain metastases). Recombinant FLAG-tagged netrin-1 served as a positive control showing higher molecular weight due to the FLAG-tag. Intensity measurements of either full length or ΔN-netrin-1 in association with alpha-tubulin loading control resulted in the depicted ratio. ImageJ software (NIH, USA) was used for quantification. (B) Corresponding cryo sections from the same tumor specimens were stained for netrin-1 by automated immunohistochemistry. Left row showing a representative tumor area, right row highlighting the nuclear compartment (scale bar left row 100 μm, scale bar right row 50 μm). (C) Corresponding qRT-PCR analyses from extracted mRNA of the same tumor specimens as assessed for protein expression levels is depicted. Netrin-1 (NTN1) expression relative to HPRT1 and G6PD1 housekeeping genes. We used a primer set overspanning intron 1 of the netrin-1 (NTN1) gene (forward primer: GCAAGCCCTTCCACTACGAC; reverse primer: CGACAGTTGAGGCAGACACCT). (TIF) [file pone.0092311.s002.tif]

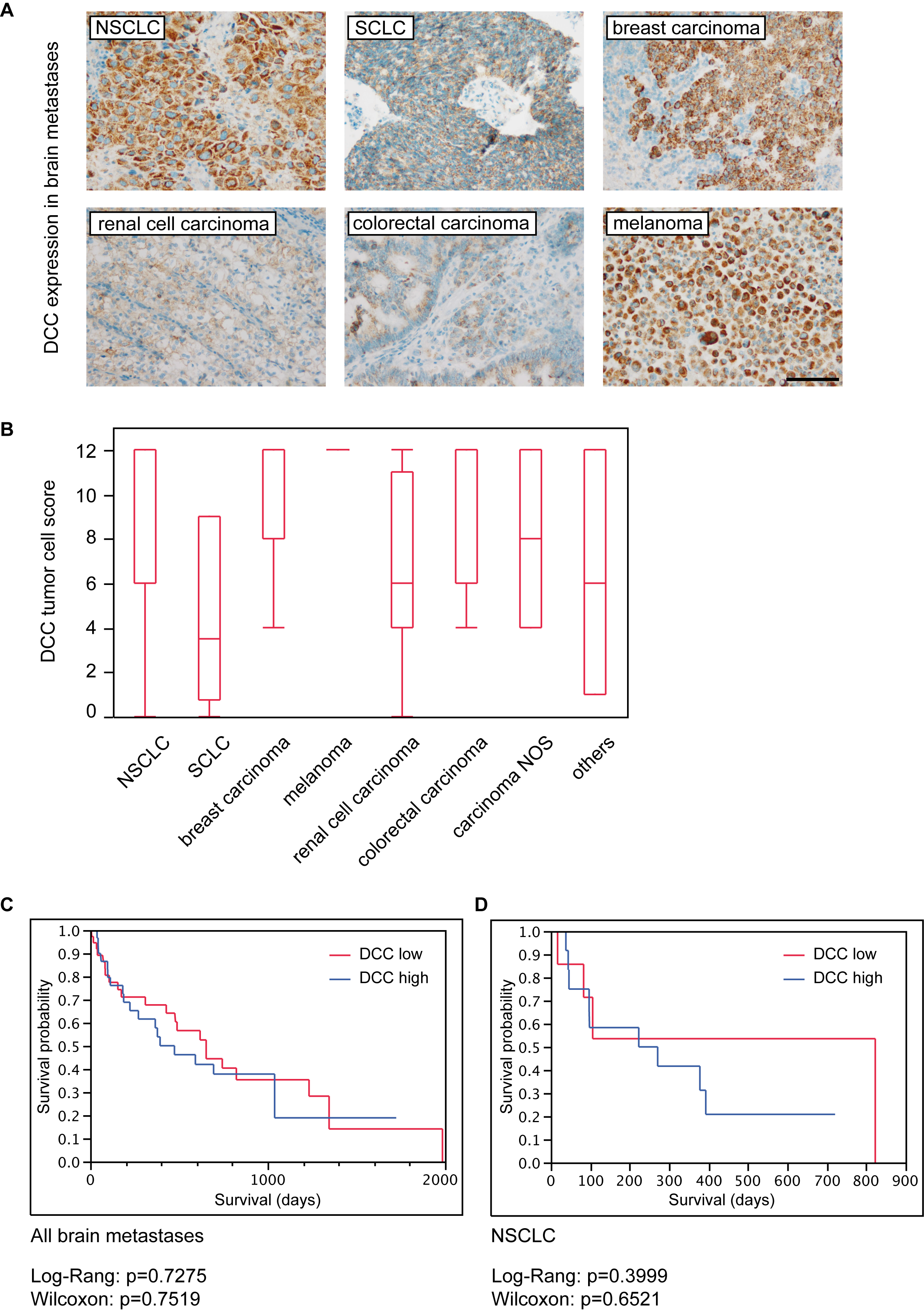

Supplement: Figure S3 — DCC expression in human brain metastases. DCC is strongly expressed in tumor cells of human brain metastases and is mainly localized in a granular pattern in cytoplasms but also on cell membranes (A; scale bar 100 μm). Box plots of DCC tumor cell scores are shown in (B) (see also Table S1). (C, D) No association of DCC tumor cell scores with patient survival neither in (C) the entire cohort nor in (D) the subentity of NSCLC was found. (TIF) [file pone.0092311.s003.tif]
