# Supplementary material for: Netrin-1 Expression Is an Independent Prognostic Factor for Poor Patient Survival in Brain Metastases
Source: PLoS One. 2014 Mar 19;9(3):e92311. doi: 10.1371/journal.pone.0092311 (PMC3960244; doi:10.1371/journal.pone.0092311)
Supplement: Table S1 — Median and mean expression levels of netrin-1 and DCC in human brain metastases. Median tumor cell expression scores of netrin-1 and DCC and percentages of netrin-1-positive tumor cell nuclei (per total tumor cell nuclei) in human brain metastases. (DOCX) [file pone.0092311.s004.docx]

| *Brain metastases* | *median netrin-1 tumor cell score* | *median DCC tumor cell score* | *mean netrin-1 nuclear expression % (SEM)* |
| --- | --- | --- | --- |
| NSCLC | 6 | 12 | 13.3 (2.9) |
| SCLC | 4 | 3.5 | 14.3 (11.2) |
| Breast carcinoma | 4 | 8 | 9.0 (2.9) |
| Melanoma | 3 | 12 | 20.2 (5.3) |
| Renal cell carcinoma | 4 | 6 | 6.0 (1.9) |
| Colon carcinoma | 4 | 6 | 14.6 (9.6) |
| Carcinoma NOS | 6 | 8 | 30.4 (10.1) |
| Others | 3.5 | 6 | 21.3 (12.1) |

**Supporting Table 1: Median and mean expression levels of netrin-1 and DCC in human brain metastases.**

Median tumor cell expression scores of netrin-1 and DCC and percentages of netrin-1-positive tumor cell nuclei (per total tumor cell nuclei) in human brain metastases.
