# Supplementary material for: Netrin-1 Expression Is an Independent Prognostic Factor for Poor Patient Survival in Brain Metastases
Source: PLoS One. 2014 Mar 19;9(3):e92311. doi: 10.1371/journal.pone.0092311 (PMC3960244; doi:10.1371/journal.pone.0092311)
Supplement: Table S2 — Multivariate survival analysis in our patient cohort excluding NSCLC. Cox hazard ratios of nuclear netrin-1, Ki67, patient age and number of brain metastases are depicted (single comparisons of diagnoses and edema scores are not shown. n/a not applicable). (DOCX) [file pone.0092311.s005.docx]

| Variable | Results of multivariate Cox regression | |
| --- | --- | --- |
|  | **p-Value** | **Hazard Ratio (95% CI)** |
|  |  |  |
| Diagnosis  Patient age  Number of brain metastases  Edema score | 0.0182  0.8143  0.0211  0.0912 | n/a  1.01 (0.93-1.09)  1.17 (1.03-1.33)  n/a |
| Ki67 (%) | 0.2142 | 1.02 (0.99-1.06) |
| Netrin-1 nuclear (%) | 0.0018 | 1.04 (1.01-1.08) |

**Supporting Table 2: Multivariate survival analysis in our patient cohort excluding NSCLC.**

Cox hazard ratios of nuclear netrin-1, Ki67, patient age and number of brain metastases are depicted (single comparisons of diagnoses and edema scores are not shown. n/a not applicable).
